# Obesity-Related Dietary Behaviors among Racially and Ethnically Diverse Pregnant and Postpartum Women

**DOI:** 10.1155/2016/9832167

**Published:** 2016-05-19

**Authors:** Ashley Harris, Nymisha Chilukuri, Meredith West, Janice Henderson, Shari Lawson, Sarah Polk, David Levine, Wendy L. Bennett

**Affiliations:** ^1^Division of General Internal Medicine, The Johns Hopkins University School of Medicine, Baltimore, MD 21205, USA; ^2^Department of Gynecology and Obstetrics, The Johns Hopkins University School of Medicine, Baltimore, MD 21205, USA; ^3^Department of Pediatrics, Division of General Pediatrics and Adolescent Medicine, The Johns Hopkins University School of Medicine, Baltimore, MD 21205, USA; ^4^Welch Center for Prevention, Epidemiology and Clinical Research, The Johns Hopkins University, Baltimore, MD 21205, USA; ^5^The Johns Hopkins Bloomberg School of Public Health, Baltimore, MD 21205, USA

## Abstract

*Introduction.* Obesity is common among reproductive age women and disproportionately impacts racial/ethnic minorities. Our objective was to assess racial/ethnic differences in obesity-related dietary behaviors among pregnant and postpartum women, to inform peripartum weight management interventions that target diverse populations.* Methods.* We conducted a cross-sectional survey of 212 Black (44%), Hispanic (31%), and White (25%) women, aged ≥ 18, pregnant or within one year postpartum, in hospital-based clinics in Baltimore, Maryland, in 2013. Outcomes were fast food or sugar-sweetened beverage intake once or more weekly. We used logistic regression to evaluate the association between race/ethnicity and obesity-related dietary behaviors, adjusting for sociodemographic factors.* Results.* In adjusted analyses, Black women had 2.4 increased odds of fast food intake once or more weekly compared to White women (CI = 1.08, 5.23). There were no racial/ethnic differences in the odds of sugar-sweetened beverage intake.* Discussion.* Compared with White or Hispanic women, Black women had 2-fold higher odds of fast food intake once or more weekly. Black women might benefit from targeted counseling and intervention to reduce fast food intake during and after pregnancy.

## 1. Introduction

Obesity is increasingly prevalent among reproductive age women [1–3] and is associated with pregnancy complications such as gestational diabetes and hypertensive disorders of pregnancy [2, 4–6]. Obesity disproportionately impacts women of racial and ethnic minorities, with highest rates in Black women, followed by Hispanic and White women [3], and potentially contributes to racial and ethnic disparities in other chronic diseases such as diabetes, hypertension, and cardiovascular disease [7–9]. Like preconception obesity, gaining excessive gestational weight disproportionately affects racial and ethnic minorities [2] and is associated with pregnancy complications and future risk of long term overweight and obesity [10, 11].

Pregnancy provides an opportunity to identify unhealthy behaviors and promote healthy eating habits, which can be sustained beyond pregnancy. Understanding racial and ethnic differences in health behaviors could inform and target future interventions. However, evidence is not yet clear as to whether the observed racial and ethnic differences in preconception obesity and gestational weight gain are associated with differences in obesity-related dietary behaviors [12–16]. Further, we found no studies specifically evaluating fast food and sugar-sweetened beverage intake in this population, two potentially modifiable dietary behaviors which are associated with obesity [17–19]. Previous studies used health behavior surveys to evaluate differences in dietary habits between Black and Hispanic women, but results did not show consistent differences between the racial and ethnic groups [12–14, 16]. The largest of these four studies excluded women with chronic medical comorbidities such as hypertension or diabetes [12] or high risk pregnancies [16]. Another large, prospective cohort of 2394 women used food frequency questionnaires to evaluate dietary differences among racial groups [15], but the study populations were primarily middle class.

Our primary objective was to address an important evidence gap: the association between race and ethnicity and differences in women's obesity-related dietary behaviors during pregnancy and after delivery, among a socioeconomically diverse group of women. Based on evidence from previous studies [1–3, 12, 14], we hypothesized that Black women would have higher odds of both fast food and sugar-sweetened beverage intake.

## 2. Methods

### 2.1. Study Design

We conducted a cross-sectional analysis using data collected in a convenience sample, using a one-time, self-administered questionnaire describing health behaviors among a sample of pregnant and postpartum women. This study was approved by the Johns Hopkins University School of Medicine Institutional Review Board.

### 2.2. Sample Population

A total of 247 English or Spanish speaking women, ≥18 years old, pregnant or within 1 year postpartum, who reported the ability to read the survey in English or Spanish, completed the survey. Women were recruited from 1 of 4 outpatient clinics (including high risk obstetrics and pediatrics practices) in 2 academic hospitals in Baltimore, Maryland, between January and April 2013. Participants completed a one-time, self-administered questionnaire at the time of their or their children's appointments. Of the 247 women, the 212 women who were identified as Black, Hispanic, or White were included in this secondary analysis. The 35 participants in the other racial categories were as follows: 4% were Asian, 0.4% were Hawaiian or Pacific Islander, 1.6% were American Indian or Native Alaskan, and 3.3% described themselves as multiethnic. The diversity of the other racial/ethnic group limited our ability to make comparative inferences about their dietary habits, and these women were thus excluded from this analysis.

Our study was performed on a convenience sample. Data was collected only from those women who were approached and agreed to participate. We did not calculate the percentage of patients approached who agreed to participate in our study or evaluate the ways in which the participating women may differ from those women that chose not to participate.

### 2.3. Measure

The survey, which included questions about sociodemographics and dietary behaviors, was adapted from validated national survey instruments [20–22]. Items on fast food were adapted from the Coronary Artery Risk Development in Adults Study: How many times in the past week did you eat out in a fast food restaurant such as McDonald's, Burger King, Wendy's, Arby's, Pizza Hut, or Kentucky Fried Chicken? (1) Never or less than once weekly, (2) 1-2 times per week, (3) more than 3 times per week but less than daily, or (4) at least daily [20]. Items related to sugar-sweetened beverages were adapted from the Behavioral Risk Factor Surveillance System: In the past 7 days, how often did you drink soda (not diet) or other sugar-sweetened beverages, like Hawaiian Punch, lemonade, or Kool-Aid? (1) Never or less than 1 can per week, (2) 1-2 cans per week, (3) more than 3 cans per week but less than daily, (4) about 1 can per day, or (5) 2 or more cans per day [21].

The final questionnaire was translated into Spanish and back translated to English. A pilot study was performed to ensure that the questionnaire met criteria for a 5th-grade literacy level, as well as culture appropriateness, ease of understanding, and quick time to completion.

### 2.4. Definition of Main Predictor Variables: Race/Ethnicity and BMI

Race and ethnicity were self-reported on the questionnaire. Participants were asked the following questions: Are you Hispanic or Latino? Which of the following best describes your race? Check all that apply: Asian, African American or Black, Caucasian/White/European American, Native Hawaiian or other Pacific Islander, American Indian/Alaska Native, or Multiethnic or mixed. We then categorized the racial and ethnic groups into African American/Black, Hispanic, Caucasian, or other races/ethnicities. As above, women reporting other racial or ethnic categories were not included in the analysis.

Preconception body mass index (BMI) was calculated based on self-reported height and preconception weight for pregnant women and current weight for postpartum women and categorized into obese (BMI ≥ 30) and nonobese (BMI < 30) [23, 24].

### 2.5. Definition of Outcomes

The primary outcomes were fast food frequency and sugar-sweetened beverage intake, both defined as less than once weekly versus once or more weekly. The rationale for these cut-points was based on the median intake in our sample. Prior studies used similar cut-points and showed that the consumption of fast food two or more times per week was associated with weight gain and insulin resistance over 15 years, when compared to those who eat fast food less than twice weekly [18]. Notably, existing literature on sugar-sweetened beverage intake demonstrated greatest risk of weight gain [25] and coronary heart disease [26, 27], with at least daily consumption of sugar-sweetened beverages. A frequency cut-point of once or greater per week was also deemed simple to assess clinically and to be potentially actionable.

### 2.6. Other Covariates

Sociodemographic variables included age, language proficiency, marital status, education, employment, and income. Age was categorized as follows: 18–24, 25–29, 30–34, and ≥35. English language proficiency was categorized as “adequate” if the respondent reported very good English language proficiency and “limited” for other responses, based on response categorization in the US Census [28]. A binary variable for marital status was created to assess differences between those who were married or living with a partner and those who were not.

Education level was divided into three variables including those with less than a high school education, those graduating high school or obtaining a GED, and those with one or more years of college. Employment was assessed and categorized into employed (full or part time), unemployed, maternity leave, home maker, disability, or student. We also assessed income and categorized the data into broad categories, as noted in Table 1. Financial strain was a separate income variable, based on participant response to the survey question, “In the past 12 months, was there ever a time when you did not have enough money to meet the daily needs of you and your family?”

Pregnancy status and medical comorbidities were binary variables based on responses to the question, “Have you been told that you have had any of these health problems? Check all that apply: overweight or obese, type 2 diabetes, gestational diabetes (diabetes in pregnancy), high blood pressure, preeclampsia or toxemia, and none of the above.” A binary variable was created for smoking, in which a yes response represents any smoking.

### 2.7. Analysis

Descriptive analyses were used to explore the data by race and obesity categories and to describe the proportion who endorsed barriers to healthy behaviors. We utilized univariate and multivariate logistic regression models to evaluate for confounders [29]. Age, marital status, English language proficiency, presence of a child under age 5 at home, and education level were included in the model based on our review of the literature. Financial strain was included as the financial variable, rather than income, due to concerns about differential bias as a result of the large percentages of Black (23%) and Hispanic (37%) participants who declined to answer the question on income.

To evaluate the role of obesity in the relationship between race/ethnicity and dietary behaviors, we assessed effect modification using stratified samples by BMI ≥ 30 and <30. The rationale was that obesity may be the result of poor dietary behaviors but obese pregnant women may be more likely to receive behavioral counseling and thus make lifestyle changes. While we do not know of any specific data examining the role of race in these behaviors, there is data demonstrating racial and cultural differences in body image [30], which could potentially lead to modification of racial differences in fast food and sugar-sweetened beverage intake, based on obesity.

We performed two sensitivity analyses. First, we limited the sample to include only the pregnant women (*n* = 179) as pregnant and postpartum women may report different behaviors. The percentage of postpartum women was so small (16%) that we were unable to compare these two groups. We compared the results in just pregnant women to the results in the entire model to assess for differences. Second, we changed the cut-point of sugar-sweetened beverage intake to assess daily not weekly intake, ≥1 versus <1 sugar-sweetened beverage daily, and reevaluated our model. This sensitivity analysis was designed to address the difference between the cut-point we utilized in our study (intake of 1 or more sugar-sweetened beverages weekly) and that used in the literature pertaining to sugar-sweetened beverages (intake of 1 or more sugar-sweetened beverages daily) [25–27].

## 3. Results

Table 1 shows the characteristics of the 212 women in our sample by race/ethnicity.

**Figure 1:**
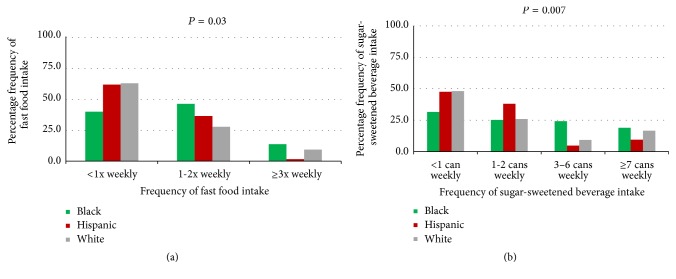
(a) Unadjusted weekly fast food intake by race/ethnicity. (b) Unadjusted weekly sugar-sweetened beverage intake by race/ethnicity.

Figure 1 shows the preadjustment frequency of fast food (Figure 1(a)) and sugar-sweetened beverage (Figure 1(b)) intake by race/ethnicity. Overall, 52% of women reported fast food intake less than once weekly or never, 39% reported intake 1-2 times weekly, and 9% reported intake ≥3 times weekly. In terms of sugar-sweetened beverage intake, the plurality (40.6%) reported less than 1 serving weekly, while 29.3% reported 1-2 servings and 14.6% reported 3–6 servings weekly.

### 3.1. Adjusted Analyses to Assess Racial and Ethnic Differences in the Odds of Fast Food and Sugar-Sweetened Beverage Intake

Table 2 shows the results of our adjusted logistic regression models. With respect to fast food intake, Black women had 2.38 times higher odds of consumption once or more weekly, when compared to White women (CI = 1.08 and 5.23). We did not detect differences in fast food frequency between Hispanic and White women (CI = 0.45 and 2.70). Women aged 30–34 had 2.6 times higher odds when compared to women 18–24 years old (CI = 1.02 and 6.62). There were no other differences in intake by age group. Women reporting financial strain had 1.4 times greater odds of fast food intake than those who did not report financial strain (CI = 1.01 and 1.93). Women who were married or lived with a partner had 0.4 reduced odds of consuming fast food (CI = 0.21 and 0.85), when compared to those without a spouse or live-in partner.

In adjusted analyses, we did not detect racial/ethnic differences in sugar-sweetened beverage intake. Compared to those without young children at home, women with a child under age 5 at home were 3.0 times more likely to drink sugar-sweetened beverages once or more weekly (CI = 1.54 and 6.00). Married women and those living with a partner had reduced odds of drinking sugar-sweetened beverages (OR = 0.30 and CI = 0.13, 0.68) compared with unmarried and single women. Lastly, women aged 35 years or older had lower odds of sugar-sweetened beverage intake when compared to women 18–24 (CI = 0.15 and 0.99).

In stratified analyses, we did not detect racial/ethnic differences in fast food intake among obese women. However, nonobese Black women had 4.66-fold greater odds of fast food intake once or more weekly, when compared to nonobese White women (CI = 1.49 and 14.5). There were no significant racial/ethnic differences in sugar-sweetened beverage intake in the stratified obese or nonobese subgroups. Results were otherwise very similar to those seen in the analysis of the entire cohort.

The first sensitivity analysis, in which we excluded postpartum women and examined the adjusted odds of fast food and sugar-sweetened beverage intake, showed that Black women had 2.6 times higher odds of fast food intake once or more weekly when compared with White women (CI = 1.10 and 6.06), confirming our findings from the entire sample. Results were also similar for the other variables (data not shown).

In the second sensitivity analysis, we assessed a daily cut-point for sugar-sweetened beverage intake, comparing the 18% of our sample that reported daily versus nondaily sugar-sweetened beverage intake. Even with this different cut-point, we confirmed a null association between race and ethnicity and the odds of drinking one or more sugar-sweetened beverages daily.

## 4. Discussion

In a cross-sectional analysis of 212 pregnant and postpartum women, 47.7% of women reported eating fast food one or more times weekly, but only 1.9% consumed it one or more times daily. In contrast, 59% reported drinking one or more sugar-sweetened beverages per week, with 15.6% drinking at least one can daily. We found significant racial and ethnic differences in fast food, but not sugar-sweetened beverage, intake. Black women had 2-fold greater odds of fast food intake once or more weekly when compared with White women. The increased strength of the association among nonobese Black women was interesting in light of previous studies demonstrating that normal and overweight women are at greater risk of excess gestational weight gain than obese women [2, 16]. This emphasizes the need for inclusion of nonobese women in discussions around dietary habits, healthy gestational weight gain, and postpartum weight loss. Our data provides new information about racial differences in dietary behaviors and highlights the need for interventions to target obesogenic dietary behaviors in pregnancy and postpartum, as failure to lose gestational weight during the first year postpartum is associated with worsened cardiovascular risk markers [31] and overweight at 15 years postpartum [32, 33].

Marriage or living with a partner was associated with reduced odds of both fast food and sugar-sweetened beverage intake [34–36]. Our finding may represent increased financial means, improved social support, or factors not measured in our study. This data adds new information to existing literature on marriage and pregnancy related health behaviors. Prior data has shown decreased use of tobacco and drugs during pregnancy [34], increased prenatal care [35], and improved pregnancy outcomes [36] among women who have a good relationship with the father of their child, when compared to those without such a relationship.

Financial strain was associated with increased odds of both fast food and sugar-sweetened beverage intake when compared with women who did not report financial strain. This finding was expected given that healthier foods are often more expensive and less available in lower income neighborhoods lacking a grocery store with healthy food options. Fast food and sugar-sweetened beverages may represent less expensive alternatives to grocery purchased food for low-income families.

The finding that women having children under age 5 in the home were more likely to drink sugar-sweetened beverages was counter-intuitive. One possible explanation might be parental fatigue, leading to higher intake of caffeinated beverages that contain sugar. Another possible explanation might be having the sugary drinks on hand for children, leading to increased intake on the part of the parent.

The findings of increased fast food intake among women 30–34 and decreased sugar-sweetened beverage intake per month among women 35 and over were unexpected. Further study is needed to confirm and further evaluate these findings.

While the majority of data on postpartum weight loss interventions have focused on middle class White women [37–40], studies in the general population have shown that culturally tailoring interventions can result in significant weight loss in low-income and racial and ethnic minority groups [41–44]. Concern exists, however, that Black women lose less weight than their White counterparts [45] and drop-out rates for all participants remain high [40, 46]. Several qualitative studies have examined barriers to, and facilitators of, healthy lifestyles in pregnant and postpartum populations, and these results should be considered when designing dietary interventions [47–49].

Our results highlight the need to address sugar-sweetened beverage intake in all pregnant and postpartum women, while specifically targeting reduction in fast food intake among Black women. A successful approach to changing high risk dietary behaviors among Black women could involve recruitment from trusted community sources, such as churches and community centers, which have been shown to be helpful in weight loss [50]. Further study would be needed to see if this improves recruitment as well as retention in interventions. Educational interventions, such as nutritional seminars or guided grocery shopping, could be practical methods to address educational barriers and enhance awareness of what constitutes a healthy diet and healthy body weight and healthy available alternatives to fast food [12, 51, 52]. Finally, cultural adaptations of dietary recommendations have resulted in weight loss among African Americans in nonpregnant populations [53, 54]. Further study is needed to determine what specific cultural adaptations would be most helpful in changing dietary habits among pregnant and postpartum women.

In addition to interventions specifically for Black women, we would also recommend broad interventions that would improve nutrition among all pregnant and postpartum women, regardless of race or ethnicity. These would include interventions to address time constraints and lack of social support [41, 55, 56], as well as family preferences. Online follow-up allows women to participate in their schedule. This format could reinforce person educational activities and serve as a forum for recipe sharing and meal planning and provide ongoing motivation and peer support [55]. Concurrent policy initiatives should be employed to complement the clinical interventions, ensuring access to healthier, more affordable foods [57–60] and decreasing access to unhealthy foods such as sugar-sweetened beverages and fast food through taxation [61] as well as insurance reimbursement for successful weight loss programs [62–65].

The major strengths of our study were in the diverse sample of Black, Hispanic, and White participants to evaluate racial and ethnic differences, while controlling for key socioeconomic variables and preconception BMI. This study has several limitations. This was a small study. Our survey was based on a convenience sample. Some groups may have been under- or overrepresented as a result of not using probability samples. We are unable to report how many women declined to participate or how those who chose to participate differ from those who did not. This can introduce bias; however, this also allowed us to evaluate real-world clinical populations. High nonresponse rates occurred with some of our key variables. Higher percentages of Hispanic (41%) and Black (6.5%) women did not answer the self-report question pertaining to preconception weight and height, as compared to 1.9% of White women. Hispanic women were also less likely to be insured or have a PCP, which may lead to a lack of knowledge of weight and preconception medical diagnoses. These results suggest that the preconception rates of overweight and obesity may be higher in both groups than reported. To address this limitation, we compared our results to national survey data on obesity for women aged 20–39 years [66] and found similar obesity rates for Black and White women but underestimated rates for Hispanic women, likely as a result of missing data. Likewise, incomplete responses to certain socioeconomic variables limited their use as covariates in our study. Notably 36.5% of Black women, 23.2% of Hispanic women, and 11.1% of White women declined to answer the question about income. We thus used financial strain as a measure of wealth in its stead.

## 5. Conclusions

We found significantly increased odds of fast food intake among pregnant and postpartum Black women when compared to White women, with an even stronger association among nonobese Black women. We found high intake of sugar-sweetened beverages among all women. These results suggest the need for nutritional counseling about fast food intake targeted at Black women, including nonobese women, and about sugar-sweetened beverage intake in all women.

## Figures and Tables

**Table 1:** Characteristics of pregnant and postpartum women by race/ethnicity^*∗*^.

	Overall *n* = 212	Black *n* = 95	Hispanic *n* = 63	Caucasian *n* = 54	*P* value
	Number (%)	Number (%)	Number (%)	Number (%)	
*Individual demographic covariates*					

Maternal age					**<0.0001**
18–24	74 (34.9)	46 (48.4)	21 (33.3)	7 (13.0)	
25–29	65 (30.7)	30 (31.6)	19 (30.2)	16 (29.6)	
30–34	36 (17.0)	9 (9.5)	12 (19.0)	15 (27.8)	
≥35	37 (17.4)	10 (10.5)	11 (17.5)	16 (29.6)	

English language proficiency^a^					**<0.0001**
Adequate	157 (74.1)	94 (98.9)	10 (15.9)	53 (100)	

Language spoken at home					**<0.0001**
English	157 (75.1)	94 (100)	10 (16.1)	53 (100)	

Marital status					**<0.0001**
Married/live-in partner	153 (68.6)	50 (52.6)	54 (85.7)	43 (79.6)	

Childcare^b^					**0.04**
Yes	43 (20.3)	46 (48.4)	16 (25.4)	24 (44.4)	
Not required	86 (40.6)	31 (32.6)	31 (49.2)	19 (35.2)	
No	81 (38.2)	18 (19.0)	14 (22.2)	11 (20.4)	

Child under 5 years old					0.28
Yes	139 (65.6)	63 (66.3)	45 (71.4)	31 (57.4)	

Education					**<0.0001**
≤grade 11	53 (25.2)	13 (13.8)	33 (53.2)	7 (13.0)	
High school/GED	73 (34.8)	45 (47.9)	19 (30.7)	9 (16.6)	
≥1-year college	84 (40.0)	36 (38.3)	10 (16.1)	38 (70.4)	

Employment					**<0.0001**
Employed full/part time	75 (36.6)	34 (37.0)	14 (23.7)	27 (50.0)	
Unemployed	52 (25.4)	31 (33.7)	12 (20.3)	9 (16.7)	
Maternity leave	12 (5.89)	10 (10.9)	0 (0.0)	2 (3.7)	
Disability	10 (4.9)	6 (6.5)	1 (1.7)	3 (5.5)	
Homemaker	46 (22.4)	6 (6.5)	30 (50.8)	10 (18.6)	
Student	10 (4.9)	5 (5.4)	2 (3.5)	3 (5.5)	

Income					**<0.0001 **
<10,000	49 (23.1)	33 (34.7)	11 (17.5)	5 (9.3)	
10,000–19,999	41 (19.3)	18 (18.9)	16 (25.4)	7 (13.0)	
20,000–34,999	23 (10.9)	13 (13.7)	7 (11.1)	3 (5.5)	
35,000–49,999	10 (4.7)	5 (5.3)	2 (3.2)	3 (5.5)	
>50,000	38 (17.9)	4 (4.2)	4 (6.3)	30 (55.6)	
Declined to answer	51 (24.1)	22 (23.2)	23 (36.5)	6 (11.1)	

Financial strain					**0.003**
Yes	92 (41.3)	46 (48.4)	29 (46.0)	12 (22.2)	
No	127 (56.9)	48 (50.5)	31 (49.2)	42 (77.8)	

*Access to care*					

Insurance					**<0.0001**
Private	51 (25.3)	14 (15.1)	5 (8.9)	32 (60.4)	
Medicaid	110 (54.5)	74 (79.6)	15 (26.8)	21 (39.6)	
Medicare	4 (2.0)	3 (3.2)	1 (1.8)	0 (0.0)	
Uninsured	37 (18.3)	2 (2.1)	35 (62.5)	0 (0.0)	

Primary care physician					**<0.0001**
Yes	131 (58.7)	69 (72.6)	12 (19.0)	45 (83.3)	
No	90 (40.4)	25 (26.3)	50 (79.4)	9 (16.7)	
Do not know	2 (0.9)	1 (1.1)	1 (1.6)	0 (0.0)	

*Medical status*					

Pregnancy status					0.1
Pregnant	179 (84.4)	83 (87.4)	48 (76.2)	48 (88.9)	
Postpartum	33 (15.6)	12 (12.6)	15 (23.8)	6 (11.1)	

Prepregnancy BMI^c^					**<0.0001**
<18.5	5 (2.3)	2 (2.1)	1 (1.6)	2 (3.7)	
18.5–24.9	50 (23.6)	17 (17.9)	9 (14.3)	24 (44.4)	
25–29.9	49 (23.1)	22 (23.2)	15 (23.8)	12 (22.2)	
30–39.9	60 (28.3)	37 (38.9)	10 (15.9)	13 (24.1)	
>40.0	15 (7.1)	11 (11.6)	2 (3.2)	2 (3.7)	
NR	33 (15.6)	6 (6.3)	26 (41.2)	1 (1.9)	

Medical comorbidities					
Any medical comorbidity	122 (57.6)	57 (60.0)	41 (65.1)	24 (44.4)	0.06
Overweight or obesity	157 (74.1)	76 (80.0)	53 (84.1)	28 (51.9)	**<0.0001 **
Obesity	108 (50.9)	54 (56.8)	38 (60.3)	16 (29.6)	**0.001**
Type II diabetes	15 (7.1)	10 (10.5)	3 (4.8)	2 (3.7)	0.21
Hypertension	24 (11.3)	12 (12.6)	4 (6.3)	8 (14.8)	0.31

Pregnancy complications					
Gestational diabetes	26 (12.3)	8 (8.4)	6 (9.5)	12 (22.2)	**0.04**

Smoke					0.9
Yes	19 (9.0)	9 (9.5)	2 (3.2)	8 (14.8)	0.09

Self-reported health status					**0.005**
Good	177 (83.5)	76 (80.0)	47 (74.6)	54 (100.0)	
Fair/poor	33 (15.6)	18 (18.9)	15 (23.8)	0 (0.0)	

Sleep quality					
Good	114 (53.8)	60 (63.2)	28 (44.4)	26 (48.1)	**0.04**

^*∗*^Numbers not adding to *N* in sample and percentages not leading to 100% are due to nonresponses.

^a^ English language proficiency defined as adequate versus not adequate.

^b^ Childcare: yes, defined as childcare other than parents obtained, not needed, defined as no children in need of childcare, and no, defined as childcare provided by parents.

^c^ Based on respondents only.

GED = graduate equivalency degree.

**Table 2:** Adjusted odds of fast food and sugar-sweetened beverage intake.

Sociodemographic covariates	Fast food intake ≥ 1 time weekly	Sugar-sweetened beverage intake ≥ 1 time weekly
	OR (CI)	OR (CI)
Maternal race, White (REF)		
Black	**2.38 (1.08, 5.23)**	0.91 (0.38, 2.17)
Hispanic	1.10 (0.45, 2.70)	0.57 (0.20, 1.49)

Maternal age, 18–24 (REF)		
25–29	1.09 (0.53, 2.25)	1.08 (0.49, 2.38)
30–34	**2.60 (1.02, 6.62)**	1.01 (0.39, 2.63)
≥35	1.03 (0.41, 2.59)	**0.38 (0.15, 0.99)**

Marital status		
Married/living with partner	**0.43 (0.21, 0.85)**	**0.30 (0.13, 0.68)**

Child under 5 years old, yes	1.20 (0.64, 2.26)	**3.04 (1.54, 6.00)**

Education		
<grade 12	1.73 (0.74, 4.07)	1.42 (0.56, 3.59)
High school graduate/GED	1.13 (0.47, 2.73)	0.52 (0.20, 1.32)
≥1-year college	1.32 (0.06, 26.9)	0.27 (0.01, 6.17)

Financial strain, yes	**1.40 (1.01, 1.93)**	1.04 (0.79, 1.38)

	*n* = 75	*n* = 75

*Subsample with BMI ≥ 30*		
Maternal race, White (REF)		
Black	0.46 (0.10, 2.00)	0.28 (0.05, 1.47)
Hispanic	0.27 (0.05, 1.33)	0.25 (0.04, 1.38)

	*n* = 99	*n* = 99

*Subsample with BMI < 30*		
Maternal race, White (REF)		
Black	**4.66 (1.49, 14.5)**	1.85 (0.57, 6.03)
White	1.39 (0.36, 5.33)	0.71 (0.18, 2.87)

Boldface denotes statistical significance.

BMI = body mass index, CI = confidence interval, GED = graduate equivalency degree, OR = odds ratio, and REF = reference.
